# Phase I study of azacitidine and oxaliplatin in patients with advanced cancers that have relapsed or are refractory to any platinum therapy

**DOI:** 10.1186/s13148-015-0065-5

**Published:** 2015-03-17

**Authors:** Apostolia M Tsimberidou, Rabih Said, Kirk Culotta, Ignacio Wistuba, Jaroslav Jelinek, Siqing Fu, Gerald Falchook, Aung Naing, Sarina Piha-Paul, Ralph Zinner, Zahid H Siddik, Guangan He, Kenneth Hess, David J Stewart, Razelle Kurzrock, Jean-Pierre J Issa

**Affiliations:** Department of Investigational Cancer Therapeutics, The University of Texas MD Anderson Cancer Center, Unit 455, 1515 Holcombe Boulevard, Houston, TX 77030 USA; Department of Internal Medicine, The University of Texas Health Science Center at Houston, 6410 Fannin Street, Suite 722, Houston, TX 77030 USA; Department of Experimental Therapeutics, Division of Cancer Medicine, The University of Texas MD Anderson Cancer Center, 1515 Holcombe Boulevard, Houston, TX 77030 USA; Department of Translational Molecular Pathology, Division of Pathology/Lab Medicine, The University of Texas MD Anderson Cancer Center, 1515 Holcombe Boulevard, Houston, TX 77030 USA; Fels Institute for Cancer Research and Molecular Biology, Temple University School of Medicine, 3307 N. Broad Street, Philadelphia, PA 19140 USA; Department of Biostatistics, The University of Texas MD Anderson Cancer Center, 1515 Holcombe Boulevard, Houston, TX 77030 USA; Division of Medical Oncology, The Ottawa Hospital Cancer Centre, University of Ottawa, 501 Smyth Road, Ottawa, ON K1H 8 L6 Canada; Department of Internal Medicine, Moores Cancer Center, University of California, 3855 Health Sciences Dr, La Jolla, CA 92093 USA

**Keywords:** DNA methylation, Platinum resistance, Copper/platinum transporter

## Abstract

**Background:**

Demethylation process is necessary for the expression of various factors involved in chemotherapy cytotoxicity or resistance. Platinum-resistant cells may have reduced expression of the copper/platinum transporter CTR1. We hypothesized that azacitidine and oxaliplatin combination therapy may restore platinum sensitivity. We treated patients with cancer relapsed/refractory to any platinum compounds (3 + 3 study design) with azacitidine (20 to 50 mg/m^2^/day intravenously (IV) over 15 to 30 min, D1 to 5) and oxaliplatin (15 to 30 mg/m^2^/day, IV over 2 h, D2 to 5) (maximum, six cycles). Platinum content, LINE1 methylation (surrogate of global DNA methylation), and CTR1 expression changes (pre- vs. post-treatment) were assessed. Drug pharmacokinetics were analyzed.

**Results:**

Thirty-seven patients were treated. No dose-limiting toxicity (DLT) was noted at the maximum dose. The most common adverse events were anemia and fatigue. Two (5.4%) patients had stable disease and completed six cycles of therapy. Oxaliplatin (D2) and azacitidine (D1 and 5) mean systemic exposure based on plasma AUC_all_ showed dose-dependent interaction whereby increasing the dose of oxaliplatin reduced the mean azacitidine exposure and vice versa; however, no significant differences in other non-compartmental modeled parameters were observed. Blood samples showed universal reduction in global DNA methylation. In tumor samples, hypomethylation was only observed in four out of seven patients. No correlation between blood and tumor demethylation was seen. The mean cytoplasmic CTR1 score decreased. The pre-dose tumor oxaliplatin levels ranged from <0.25 to 5.8 μg/g tumor. The platinum concentration increased 3- to 18-fold. No correlation was found between CTR1 score and oxaliplatin level, which was found to have a trend toward correlation with progression-free survival.

**Conclusions:**

Oxaliplatin and azacitidine combination therapy was safe. CTR1 expression was not correlated with methylation status or tissue platinum concentration.

**Electronic supplementary material:**

The online version of this article (doi:10.1186/s13148-015-0065-5) contains supplementary material, which is available to authorized users.

## Background

Platinum compounds are known to have a wide range of antitumor activity both clinically and pre-clinically [1]. However, certain tumors either are initially refractory to platinum treatment or subsequently develop resistance. For instance, oxaliplatin (a third-generation platinum compound) is recommended as a frontline therapy for colorectal cancer (CRC), but patients eventually experience resistance to oxaliplatin followed by subsequent relapse and death.

Resistance to platinum-based chemotherapy can be caused by DNA hypermethylation, a critical epigenetic process in cancer progression. The hypermethylation of specific genes by DNA methyltransferase promotes tumorigenesis [2-5] and chemotherapy resistance [6]. Although many factors contribute to chemotherapy resistance [7], flattening of the dose-response curve at higher drug doses suggests that a deficiency of certain cell-specific factors may play a significant role in decreasing cytotoxicity [8]. For instance, platinum-resistant cells have been shown to have hypermethylation of the MLH1 mismatch repair gene that is important in triggering platinum cytotoxicity [9] or a pleiotropic reduction in transporters [10,11] that is potentially reversible by a DNA methyltransferase inhibitor such as decitabine [12]. In fact, expression of the copper transporter CTR1, a main contributor to cellular platinum uptake [13], rapidly decreases once CTR1 is exposed to platinum compounds, thereby reducing platinum influx [12]. In addition, in a phase I clinical trial of the hypomethylating agent decitabine in patients with refractory solid tumors and lymphomas, administration of decitabine significantly increased CTR1 expression [14].

Since the CpG island methylator phenotype was discovered in 1999 [15], it has been extensively used in assessing patients with CRC [16,17]. The role of *de novo* methylation in cancer was validated by comparing colon cancer tumor tissue with matched normal colon tissue; the cancer cells were found to be dependent on DNA methylation-mediated epigenetic silencing of selected genes. These data suggested that driver epigenetic events are associated with cancer cell survival and can represent potential targets for therapy [18]. As innovative immunomodulation is being increasingly used in targeted therapy, the role of hypomethylation in immunomodulation is also evolving. Several investigators have demonstrated that DNA methylation plays a major role in the expression of various cancer tissue antigens and immunomodulatory checkpoints [19-21].

The hypomethylating agent azacitidine, a DNA methyltransferase inhibitor compound approved for the treatment of myelodysplastic syndrome, is known to have two main mechanisms of antineoplastic activity: cytotoxicity, resulting from its incorporation into RNA and DNA, and DNA hypomethylation, restoring normal growth control and differentiation in hematopoietic cells. The induction of DNA hypomethylation appears to require lower doses of azacitidine than does cytotoxicity [22] and may modulate the resistance mechanisms in patients with platinum-refractory advanced solid tumors; in fact, azacitidine was shown to enhance the sensitivity of platinum-resistant ovarian cancer cells to carboplatin [23].

We hypothesized that azacitidine would restore sensitivity to oxaliplatin in patients with platinum-refractory/resistant cancer. Oxaliplatin, which is comprised of an organoplatinum complex in which the platinum atom is complexed with the 1,2-diaminocyclohexane carrier ligand and with an oxalate ligand [24,25], has a different spectrum of activity and low cross-resistance with cisplatin [26]; a favorable toxicity profile, including minimal renal and auditory toxicity [25,27]; and clinical antitumor activity in a broad spectrum of tumor types. The selection of oxaliplatin was also based on the Food and Drug Administration-approved indications of this drug in advanced CRC and on empirical data demonstrating its antitumor activity in breast cancer, advanced carcinoma of the stomach, esophageal cancer, germ cell tumor, non-Hodgkin’s lymphoma, non-small cell lung cancer, and ovarian cancer.

The primary objectives of this phase I study were (a) to determine the maximum tolerated dose of an azacitidine and oxaliplatin combination regimen in patients with advanced solid tumors or lymphomas relapsed or refractory to any platinum compound and (b) to define the pharmacokinetics of azacitidine and oxaliplatin. The secondary objectives were for patients treated in the expansion phase of this study: (a) to assess the CTR1 score; (b) to assess changes in global DNA methylation; (c) to measure changes in oxaliplatin levels in tumor biopsy samples taken before and after the first cycle of azacitidine plus oxaliplatin therapy; and (d) to correlate results of the pharmacokinetic studies of azacitidine and oxaliplatin with changes in CTR1, changes in global DNA methylation, and changes in oxaliplatin levels in tissue biopsies of patients treated in the expansion phase of this study.

## Results

### Patients

A total of 41 patients were screened and 37 patients were treated. Two patients did not meet the study inclusion criteria, and two patients did not receive the study treatment because of consent withdrawal and rapidly worsening performance status, respectively. Patients’ baseline characteristics are shown in Table 1. The most common tumor type was CRC (62%), and the median number of prior systemic therapies was 4 (range, 1 to 10). The median time between prior platinum treatment and the first day on trial was 4.9 months (range, 0.7 to 56). The median number of cycles received was 2 (range, 1 to 6). Two patients received the maximum of six cycles. The remaining patients discontinued treatment because of disease progression (*n* = 35). The study was terminated early because of limited funding for tumor biopsies following the recommendation of The Cancer Therapy Evaluation Program. Therefore, only 7 of 16 patients enrolled in the expansion phase had tumor biopsies.

**Table 1:** **Patient baseline characteristics (** ***n*** **= 37)**

**Clinical characteristic**	**Number of patients (%)**
Sex	
Men	18 (49)
Women	19 (51)
Age (years)	
Median (range)	58 (31 to 79)
Race	
White	26 (70)
African-American	8 (22)
Asian	3 (8)
No. of prior therapies	
Median (range)	4 (1 to 10)
Time from diagnosis to the first cycle (months)	
Median (range)	35.5 (4 to 121)
ECOG	
0	4 (11)
1	33 (89)
RMH score	
0 to 1	28 (76)
2 to 3	9 (24)
Tumor types	
CRC	23 (62)
Gynecological	4 (11)
Lung	4 (11)
Head and neck	3 (8)
Other^a^	3 (8)

ECOG, Eastern Cooperative Oncology Group; RMH, Royal Mandersen Hospital; CRC, colorectal cancer. ^a^Cholangiocarcinoma (*n* = 1), breast cancer (*n* = 1), and esophageal cancer (*n* = 1).

### Dose escalation and expansion phase

Three patients were treated at each dose level (1 to 5), and no dose-limiting toxicity (DLT) was noted (Table 2). At dose level 6, the maximum level tested, six patients were enrolled and no DLT was reported. Therefore, dose level 6 was used in the expansion phase. Sixteen patients were treated in the expansion phase. Overall, 22 patients were treated at the maximum dose level tested.

**Table 2:** **Dose escalation cohort and distribution of patients (** ***n*** **= 37)**

**Dose level**	**Number of patients treated**	**Azacitidine (mg/m** ^**2**^ **)**	**Oxaliplatin IV (mg/m** ^**2**^ **/day)**	**DLT**
1	3	20	15	0
2	3	20	22.5	0
3	3	20	30	0
4	3	25	30	0
5	3	40	30	0
6	6	50	30	0
Expansion (dose level 6)	16	50	30	0

DLT, dose-limiting toxicity.

### Toxicity

Overall, no DLT was reported. Adverse events are summarized in Table 3. Twenty-eight (76%) patients developed adverse events > grade 1. The most common non-hematological adverse events were fatigue (*n* = 17, 46%), nausea (*n* = 13, 35%), and vomiting (*n* = 12, 32%); the most common hematological events were anemia (*n* = 18, 49%) and thrombocytopenia (*n* = 15, 41%). No grade 4 or 5 adverse events were reported. Overall, 21 grade 3 adverse events were noted in 14 (38%) patients. The most common grade 3 adverse events were anemia (*n* = 4, 11%) and nausea (*n* = 4, 11%). Peripheral neuropathy was noted in six patients (16%) (grade 1, *n* = 5; grade 2, *n* = 1).

**Table 3:** **Summary of adverse events at least possibly related to the treatment reported by ≥10% of patients overall (safety population)**

**Toxicity**	**Grade 1**	**Grade 2**	**Grade 3**	**Total (%)**
Non-hematological				
Fatigue	5	10	2	17 (46)
Nausea	9	1	4	13 (35)
Vomiting	5	4	3	12 (32)
Constipation	4	5		9 (24)
Hyponatremia	4		3	7 (19)
Hypomagnesemia	7			7 (19)
Hypokalemia	4	2	1	7 (19)
Hypocalcemia	5	2		7 (19)
Peripheral neuropathy	5	1		6 (16)
Creatinine increase	4	2		6 (16)
Alkaline phosphatase increase	2	4		6 (16)
Dyspnea	5			5 (14)
Elevated AST/ALT	2	2	1	5 (14)
Hypoalbuminemia	3	1		4 (11)
Hyperkalemia	3	1		4 (11)
Diarrhea	4			4 (11)
Hematological				
Anemia	2	12	4	18 (49)
Thrombocytopenia	13	2		15 (41)
Leukopenia	6	4		10 (27)
Lymphocytopenia	3	3	2	8 (22)
Neutropenia	4	1	1	6 (16)

### Pharmacokinetics and platinum concentration

**Figure 1:**
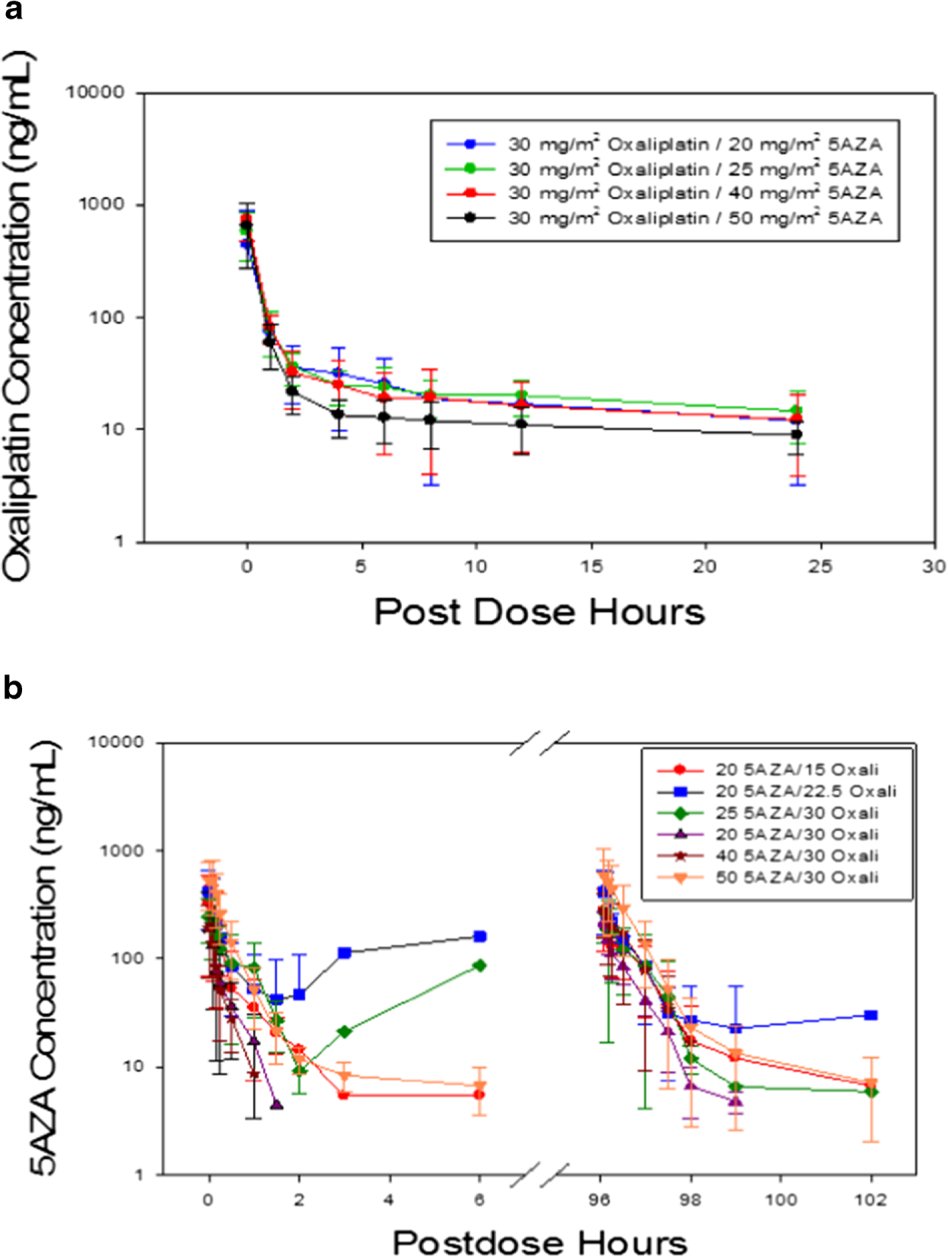
**Plasma concentrations of oxaliplatin and azacitidine. (a)** Pharmacokinetic results for oxaliplatin. **(b)** Pharmacokinetic results for azacitidine.

The plasma concentrations of oxaliplatin and azacitidine are summarized in Figure 1a,b. Briefly, the concentration of both oxaliplatin and azacitidine dropped rapidly after infusion. The concentration-time curves for azacitidine demonstrated a negligible difference between treatment days 1 and 5 at any dose level.

The pharmacokinetic data for all dose levels of oxaliplatin are summarized in Table 4. At the maximum dose level tested, the oxaliplatin *C*_max_ was 650 ng/ml, the *t*_1/2_ was 16.5 h, the clearance was 24.5 l/h/m^2^, the volume of distribution was 554 l/m^2^, and the AUC_0-inf_ was 1,456 h-ng/ml.

**Table 4:** **Mean (± SD) pharmacokinetic parameter estimates for oxaliplatin (day 2) using non-compartmental analysis**

**Oxaliplatin (mg/m** ^**2**^ **)**	**20/15 (** ***n*** **= 3)**	**20/22.5 (** ***n*** **= 3)**	**20/30 (** ***n*** **= 3)**	**25/30 (** ***n*** **= 3)**	**40/30 (** ***n*** **= 3)**	**50/30 (** ***n*** **= 22)**
Visit	C1D2	C1D2	C1D2	C1D2	C1D2	C1D2
AUC_all_ (h-ng/ml)	704.3 (228.5)	1,178.4 (238.0)	1,149.0 (930.3)	1,369.1 (556.1)	1,543.5 (502.1)	1,286.3 (627.0)
AUC_inf_ (h-ng/ml)	1,003.5 (211.1)	1,359.3 (167.1)	1,411.6 (1,065.1)	1,919.4 (923.3)	1,867.0 (387.0)	1,455.9 (657.6)
*C*_max_ (ng/ml)	337.3 (141.3)	573.5 (131.1)	446.9 (447.8)	582.6 (270.3)	751.7 (275.5)	649.4 (384.5)
*t*_1/2_ (h)	38.9 (31.9)	17.9 (7.7)	18.6 (11.9)	23.7 (10.2)	23.3 (15.6)	16.5 (7.1)
CL (l/h/m^2^)	15.5 (3.7)	16.7 (2.0)	29.1 (15.7)	19.2 (11.4)	16.6 (3.9)	24.5 (10.2)
*V*_d_ (l/m^2^)	829.2 (580.0)	443.5 (232.2)	794.5 (703.8)	587.5 (240.1)	613.8 (541.2)	554.0 (250.7)

*C*_max_, maximum concentration; AUC, area under the curve; *t*_1/2_, half-life of elimination; *V*_d_, volume of distribution; CL, clearance.

The pharmacokinetic data for all dose levels of azacitidine are also summarized in Table 5. On days 1 and 5, at the maximum dose level tested, the azacitidine *C*_max_ was 581 and 638 ng/ml, the *t*_1/2_ was 0.67 and 0.42 h, the clearance was 178 and 156 l/h/m^2^, the volume of distribution was 121 and 94 l/m^2^, and the AUC_0-inf_ was 412 and 432 h-ng/ml, respectively.

**Table 5:** **Mean (± SD) pharmacokinetic parameter estimates for azacitidine (days 1 and 5) using non-compartmental analysis**

**Azacitidine (mg/m** ^**2**^ **)**	**20/15 (** ***n*** **= 3)**		**20/22.5 (** ***n*** **= 3)**		**20/30 (** ***n*** **= 3)**		**25/30 (** ***n*** **= 3)**		**40/30 (** ***n*** **= 3)**		**50/30 (** ***n*** **= 22)**	
Visit	C1D1	C1D5	C1D1	C1D5	C1D1	C1D5	C1D1	C1D5	C1D1	C1D5	C1D1	C1D5
AUC_all_ (h-ng/ml)	216.4 (157.1)	193.1 (108.2)	428.1 (364.0)	317.1 (90.7)	112.5 (118.8)	143.7 (46.8)	210.1 (149.7)	207.7 (146.0)	97.5 (66.7)	205.9 (128.8)	404.5 (191.5)	426.5 (262.5)
AUC_inf_ (h-ng/ml)	220.7 (157.6)	196.0 (108.7)	2,328.0 (3,647.5)^a^	356.6 (147.7)	125.3 (108.1)	145.5 (47.1)	216.1 (147.2)	214.3 (144.8)	100.5 (67.1)	211.8 (131.8)	412.1 (195.7)	431.5 (261.7)
*C*_max_ (ng/ml)	327.0 (258.8)	333.5 (84.0)	427.2 (223.8)	452.4 (214.3)	206.5 (208.6)	214.1 (61.5)	251.9 (116.1)	273.2 (132.0)	189.6 (122.2)	289.0 (138.1)	581.1 (284.8)	637.7 (428.6)
*t*_1/2_ (h)	0.54 (0.37)	0.35 (0.18)	10.89 (18.25)	1.27 (1.45)	0.54 (0.44)	0.34 (0.04)	0.33 (0.15)	0.72 (0.37)	0.16 (0.07)	0.27 (0.04)	0.67 (1.03)	0.42 (0.09)
CL (l/h/m^2^)	167.1 (170.5)	136.7 (97.0)	62.7 (53.9)	62.9 (25.4)	239.3 (139.2)	146.1 (40.0)	168.6 (126.7)	150.0 (74.6)	616.6 (525.5)	253.0 (165.5)	177.7 (155.9)	156.2 (80.7)
*V*_d_ (l/m^2^)	92.3 (58.9)	61.1 (35.4)	78.6 (54.4)	85.4 (69.0)	226.5 (253.4)	71.6 (19.0)	70.3 (35.7)	132.2 (62.5)	105.8 (37.0)	95.6 (57.3)	120.6 (112.0)	94.0 (50.7)

^a^Median = 259.2. *C*_max_, maximum concentration; AUC, area under the curve; *t*_1/2_, half-life of elimination; *V*_d_, volume of distribution; CL, clearance.

Total platinum levels in snap-frozen tumor samples were determined pre- and post-treatment in seven patients treated in the expansion phase. The pre-dose platinum levels ranged from <0.25 to 5.8 μg/g tumor (median, 0.59). The platinum concentration increased 3- to 18-fold (median, 3.8-fold) in five post-dose tumor samples, but it remained unchanged in the two tumor samples with the highest pre-dose level.

### Methylation studies

**Figure 2:**
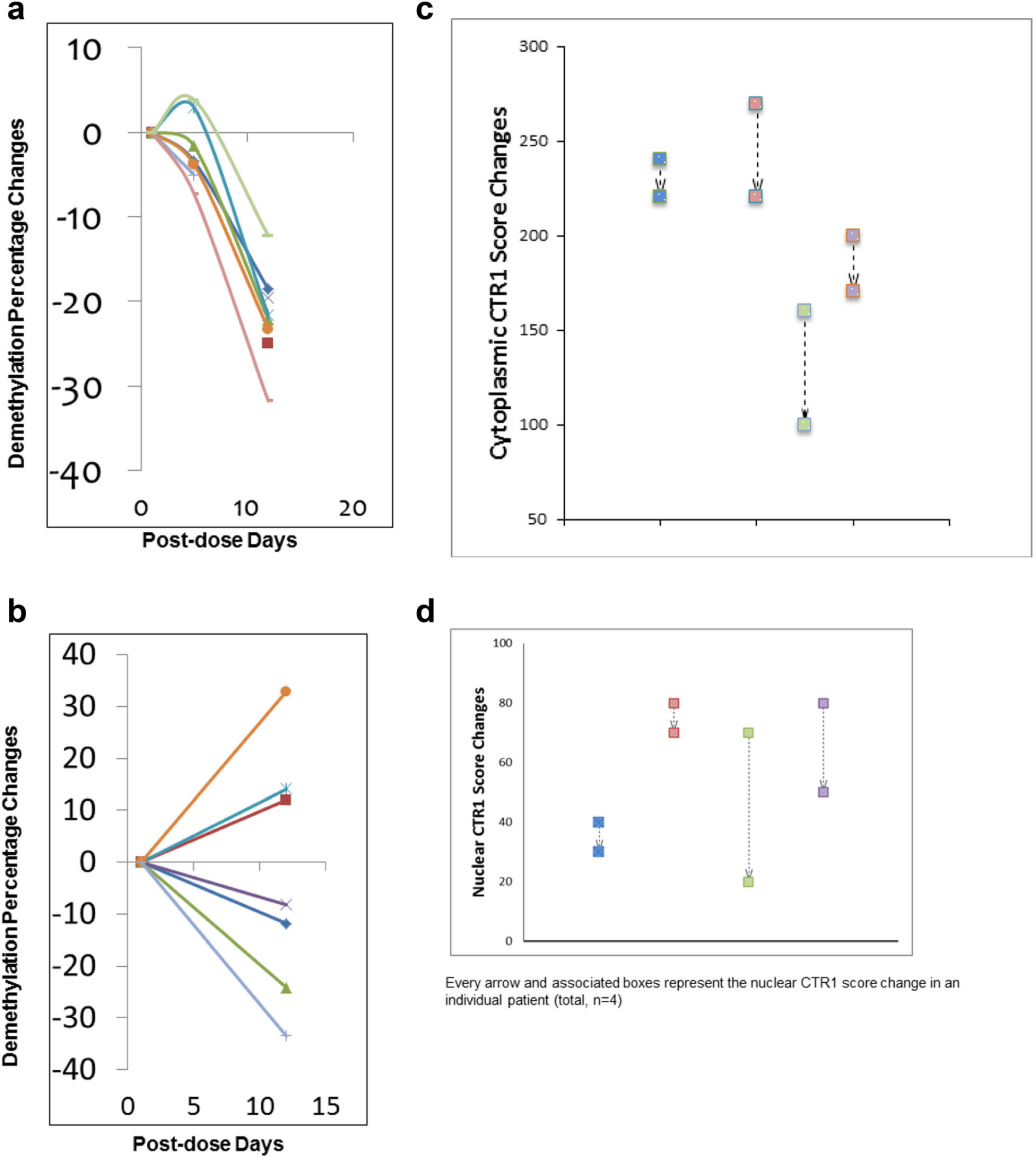
**Methylation changes and cytoplasmic and nuclear CTR1 scores pre- and post-treatment. (a)** Demethylation changes in blood. **(b)** Demethylation changes in tumor. **(c)** Cytoplasmic CTR1 score changes in tumor. **(d)** Nuclear CTR1 score changes in tumor.

Methylation studies were done using specimens from patients treated in the expansion phase. Overall, 15 biopsy samples from 8 patients were available for methylation studies. Three of these specimens were necrotic and one specimen had no tumor cells identified. Paired comparison of methylation studies (post- vs. pre-treatment) in tumor tissues was possible in seven patients. Paired comparison of methylation studies (post- vs. pre-treatment) in blood was possible in nine patients. All blood samples showed reduction in global DNA methylation from pre- to post-treatment (median, −22%; range, −32%, −12%) (Figure 2a). However, tumor samples showed mixed DNA methylation response to treatment (median, −8%; range, −34%, +33%), with a mean difference of −21.8 (95% confidence interval (CI) [18] = −26.4, −17.1; *P* < 0.0001; Figure 2b). Overall, four out of seven patients showed evidence of demethylation in the tumor. For example, one patient had a reduction in global tumor DNA methylation of −34%, but another patient had an increase in global tumor DNA methylation of +33% post-treatment. No significant correlation was seen between methylation changes and tumor tissue platinum content, and there was no correlation between demethylation in blood and demethylation in tumor tissues.

### CTR1 scores and Ki-67

Of 15 available tissue specimens (expansion phase), 5 were not evaluable for CTR1 score and Ki-67 assessment because of the absence of tumor cells. Paired comparison of CTR1 scores and Ki-67 levels (post- vs. pre-treatment) was available in four patients. The cytoplasmic and nuclear CTR1 scores pre- and post-treatment are summarized in Figure 2c,d. Briefly, the mean pre-treatment and post-treatment cytoplasmic CTR1 scores were 217.5 (range, 160 to 270) and 177.5 (range, 100 to 240), respectively, which represent a statistically significant decrease of −40 (95% CI: −69.1, −10.9; *P* = 0.022). There was also a decrease in the mean nuclear CTR1 score from pre- to post-treatment; however, it did not reach statistical significance (67.5 vs. 42.5 (−25.0; 95% CI: −55.5, +5.5; *P* = 0.08)). The average Ki-67 levels were 77.5% and 76% pre- and post-treatment, respectively. No significant correlation was seen between CTR1 scores, methylation studies, and tumor tissue platinum content. However, there was a borderline correlation between the nuclear CTR1 score and the Ki-67 changes (*P* = 0.05).

### Antitumor activity

Overall, 36 (97%) patients had response assessment after two cycles, and 13 (36%) patients had stable disease. No objective response (complete remission or partial remission) was noted. Two patients had stable disease for six cycles. The characteristics of patients with stable disease are summarized in Additional file 1: Table S1. The mean progression-free survival (PFS) duration was 2.5 months (range, 1 to 6). There was no evident correlation between PFS and the CTR1 scores. There was a trend toward correlation between the change in oxaliplatin content and PFS (Spearman *r* = 0.71, *P* = 0.07).

## Discussion

This phase I clinical trial evaluated the safety, pharmacokinetics, and pharmacodynamics of oxaliplatin and azacitidine combination therapy in patients with advanced cancer relapsed/refractory to prior platinum therapy. Patients had advanced-stage disease and were heavily pre-treated (median number of prior therapies, 4). Overall, this regimen was very well tolerated. The most common grade 3 adverse events (fatigue, cytopenia, nausea) were expected given the adverse events previously reported with each drug separately. Surprisingly, in the current study, the incidence of peripheral neuropathy was very low (16%, all grade 1 to 2; no patient had neuropathy ≥ grade 3). Other investigators have reported a high incidence of peripheral neuropathy with oxaliplatin treatment (40% to 50% for all grades; 10% to 20% for grade ≥3) [28].

The lower incidence of peripheral neuropathy in our study may be attributed to the divided dose of oxaliplatin given over 4 days compared to a higher dose given once every 2 to 3 weeks. The use of this oxaliplatin regimen was based on our design of the OFAR (oxaliplatin, fludarabine, ara-C, rituximab) regimen for patients with aggressive chronic lymphocytic leukemia/Richter syndrome [29]. With the OFAR regimen, the incidence of grade 1 to 2 neuropathy was 8% (4 of 50 patients) and no grade 3 to 4 neuropathy was noted. These protocols followed the example of the prolonged infusion of anthracyclines, which was associated with less cardiotoxicity compared to their bolus administration [30-32]. Whether this oxaliplatin infusion regimen, which seems to have a better toxicity profile, could affect the efficacy of the treatment needs further investigation.

The pharmacokinetic data for oxaliplatin showed first-order kinetics of elimination. Compared to traditional infusion (once every 2 to 3 weeks) [33], the current infusion regimen of oxaliplatin was associated with lower *C*_max_ (mean, 557 vs. 877 ng/ml) and lower AUC_inf_ (mean, 1,503 vs. 9,907 ng-h/ml) values. The major reason for the AUC and *C*_max_ difference is mainly due to the relative lower single dose. It is possible that the neurotoxicity may have been related to decreased peak drug concentrations. Similarly, the pharmacokinetics of azacitidine showed first-order kinetics of elimination, and these data were similar on day 1 (prior to oxaliplatin administration) and day 5 (after oxaliplatin administration), suggesting that oxaliplatin did not interfere with the metabolism of azacitidine and that there was no self-induction of azacitidine metabolism.

The mean systemic exposure of oxaliplatin (day 2) and azacitidine (days 1 and 5) based on plasma AUC_all_ values resulted in a dose-dependent trend. Although when the same dose of oxaliplatin was combined with increasing doses of azacitidine (from 25 to 50 mg/m^2^), a reduction in mean oxaliplatin exposure was observed, no significant differences in the other non-compartmental modeled parameters estimated were observed.

In the current study, hypomethylation was universal in the peripheral blood samples of patients, but it was not consistently observed in patients’ tumor tissues, as also seen in a previous study using decitabine in solid tumors [14]. The observed lack of demethylation has two possible explanations. First, azacitidine-induced DNA demethylation requires incorporation into DNA and thus is limited to cells in S phase. Given the short half-life of the drug, tumors with a low proliferation index might not show much demethylation. Second, clonal shifts confound DNA methylation analysis in tumors. Some cancers have profound loss of LINE1 methylation at baseline [34]. If the post-treatment biopsy contains fewer tumor cells and more normal cells than the pre-treatment biopsy, the apparent effect would be gain of LINE1 methylation, as observed here in some patients. Despite this caveat, the overall net effect was consistent with demethylation after azacitidine exposure.

The hypomethylating agent decitabine has previously been shown as a single agent to decrease DNA methylation and augment CTR1 expression in tumors with initially low CTR1 expression (immunohistochemistry (IHC) score <150) [14]. In our study, no patient had a baseline IHC score <150 (mean, 217.5; range, 160 to 270). It has been shown that the use of a platinum compound decreases CTR1 expression [12] and causes secondary platinum resistance [35]. It is unknown how oxaliplatin alone would have impacted CTR1 expression in our patients. It will require additional testing to assess whether decrease in CTR1 expression would have been much greater with oxaliplatin alone, with some mitigation by azacitidine of the impact of oxaliplatin. Whether higher doses of azacitidine would increase CTR1 expression needs further investigation; however, the effect of the dose was not seen with decitabine. Furthermore, no correlation was noted between the methylation status and CTR1 expression, which is in line with previously reported data on decitabine showing that CTR1 expression was mediated by methylation-independent mechanisms [14].

The clinical application of CTR1 expression and response to platinum agents is still evolving. In patients with non-small cell lung cancer treated with neo-adjuvant platinum-based chemotherapy, higher CTR1 expression was associated with better response to platinum compounds, but the intensity of CTR1 expression did not correlate with tumor tissue platinum concentration [36]. These findings are compatible with our observation that changes in tumor platinum concentration were not correlated with changes in CTR1 expression and that higher tumor platinum concentrations (compared to baseline) were associated with a trend toward longer PFS (*P* = 0.07).

## Conclusions

The combination of continuous-infusion oxaliplatin (days 1 to 5) and azacitidine (days 2 to 5) had a very safe toxicity profile. The most common adverse events were anemia and fatigue. Two of 37 (5.4%) heavily pre-treated patients had stable disease and completed six cycles of therapy. Oxaliplatin (D2) and azacitidine (D1 and 5) mean systemic exposure based on plasma AUC_all_ showed dose-dependent interaction whereby increasing the dose of oxaliplatin reduced the mean azacitidine exposure and vice versa. Blood samples showed universal reduction in global DNA methylation. In tumor samples, hypomethylation was observed in four of seven patients. No correlation between blood and tumor demethylation was seen. The mean cytoplasmic CTR1 score decreased. The pre-dose tumor oxaliplatin levels ranged from <0.25 to 5.8 μg/g tumor. The platinum concentration increased 3- to 18-fold. Further studies with higher doses, different schedules of azacitidine and oxaliplatin, and control arm of single-agent oxaliplatin would provide additional information regarding the effect of azacitidine on global methylation and CTR1 expression and their association with clinical outcomes.

## Methods

### Patient eligibility

Eligible patients had a histologically confirmed advanced malignancy that was metastatic or unresectable and for which standard curative or palliative measures were not expected to increase survival by at least 3 months. In addition, the patients’ cancers were relapsed or refractory to any platinum compound. Platinum-refractory disease was defined as disease that did not respond to a platinum compound-containing regimen or that recurred after treatment with a platinum compound-containing regimen. At least one prior chemotherapy regimen was required, and patients had to be ≥6 weeks beyond treatment with a nitrosourea compound or mitomycin-C and ≥4 weeks beyond any other chemotherapy or radiotherapy and recovered to ≤ grade 1 toxicity after any treatment-limiting toxicity of prior therapy. Patients were required to have acceptable clinical functions, including an Eastern Cooperative Oncology Group performance status ≤2 (Karnofsky score >60%) and normal organ and marrow functions, as defined by leukocytes ≥4,000/μl, absolute neutrophil count ≥1,500/μl, platelets ≥100,000/μl, total bilirubin ≤1.0 mg/dl, aspartate transaminase/alanine transaminase ≤3× the institutional upper limit of normal, serum creatinine ≤2.0 mg/dl, and international normalized ratio (INR) ≤1.75 (per institutional guideline). All male and female subjects of childbearing potential were educated about practicing effective contraception during the study and were willing and able to continue contraception for 3 months after their last dose of study treatment.

All patients provided written informed consent, stating that they were aware of the experimental nature of the study. This clinical trial was conducted with the approval of and in accordance with the guidelines of The University of Texas MD Anderson Cancer Center Institutional Review Board and The Cancer Therapy Evaluation Program at the National Cancer Institute (www.clinicaltrials.gov, NCT01039155).

### Treatment planning

In the dose escalation phase, patients were treated with azacitidine at 20 to 50 mg/m^2^/day intravenously (IV) over 15 to 30 min on days 1 to 5 and with oxaliplatin at 15 to 30 mg/m^2^/day IV over 2 h on days 2 to 5 (six dose levels; Table 2). This phase was followed by an expansion phase in which patients with CRC were treated with the maximum tolerated dose (MTD). Patients were treated with six cycles of therapy, unless they withdrew consent or experienced severe adverse events or progressive disease.

### Pharmacokinetics

The pharmacokinetics of azacitidine was assessed during cycle 1 (days 1 and 5) to permit correlation of pharmacokinetic parameters with hypomethylation effects. Blood samples (10 ml per sample) were to be collected at the following time points: immediately prior to azacitidine infusion, immediately after infusion, and at 5, 10, 15, 30, 60, and 90 min and 2, 3, and 6 h after infusion. Samples were assayed for azacitidine using an ultra-performance liquid chromatography/electrospray mass spectrometry method following isolation of the drug from plasma. Compartmental and non-compartmental modeling were used to derive pharmacokinetic parameters, including *C*_max_ (maximum concentration), AUC (area under the curve), *t*_1/2_ (half-life of elimination), *V*_d_ (volume of distribution), and CL (clearance).

Oxaliplatin pharmacokinetics were assessed on day 2 during cycle 1 to permit correlation of pharmacokinetic parameters with toxicity and treatment outcome. Blood samples (10 ml per sample) were collected immediately prior to oxaliplatin infusion, immediately after infusion, and at 1, 2, 4, 6, 8, 12, and 24 h after infusion. Samples were assayed for total and unbound platinum using either inductively coupled plasma mass spectrometry or atomic absorption spectroscopy methods following isolation of the drug from plasma. Compartmental and non-compartmental models were used to derive pharmacokinetic parameters, including *C*_max_, AUC, *t*_1/2_, *V*_d_, and CL.

### Pharmacodynamics

Tumor core biopsies were performed within 2 weeks before starting azacitidine and on day 12 (±1 day) of cycle 1 in the expansion phase.

#### Measurement of oxaliplatin levels in tissue

The tumor tissue was frozen and stored in cryogenic storage tubes before being analyzed for total platinum content using inductively coupled plasma mass spectrometry. If a sufficient amount of the sample remained following this analysis, the tissue was subjected to homogenization and extraction with 0.2% formic acid and acetonitrile for analysis by ultra-performance liquid chromatography/mass spectrometry for oxaliplatin content.

#### Measurement of DNA methylation

DNA was extracted and treated with bisulfite. A LINE1 assay [37] coupled with pyrosequencing [38] was used to determine global DNA methylation.

#### Immunohistochemical analysis

Five-micrometer-thick formalin-fixed and paraffin-embedded tumor tissue sections were deparaffinized and hydrated. Sections were stained using mouse antibodies for CTR1 (polyclonal; dilution 1:400; 90 min of incubation at room temperature; GeneTex Inc., San Antonio, TX, USA) and Ki-67 (monoclonal, clone MIB1; dilution 1:200; 90 min of incubation at room temperature; Dako Inc., Carpinteria, CA, USA). Cytoplasmic CTR1 was quantified using a four-value intensity score (0 to 3+). The cytoplasmic expression score (range, 0 to 300) was obtained by multiplying the intensity score and the percentage of tumor cell staining. Nuclear CTR1 and Ki-67 expressions were reported as the percentage of positive nuclei among tumor cells assessed. Changes in these scores and in the number of mitoses per high-power field, percentage of necrosis, were calculated by subtracting values at baseline from day 12 values. (Baseline values of 0 precluded calculation of percent changes.)

### Statistical considerations

A ‘3 + 3’ dose escalation study design was used. The MTD was defined as the highest dose at which six subjects were treated with at most one subject experiencing a DLT. DLT was defined as any toxicity ≥ grade 3 according to National Cancer Institute Common Terminology Criteria for Adverse Events, Version 4.0; ≥grade 3 nausea/vomiting and diarrhea for >3 days despite supportive care; or febrile neutropenia of any duration. An asymptomatic grade 3 increase in aspartate transaminase, alanine transaminase, lipase, or amylase levels lasting ≤7 days was not considered a DLT. Alopecia, grade 3 fatigue lasting ≤7 days, and grade ≥3 thrombocytopenia or neutropenia lasting ≤7 days were not considered DLTs. Once treatment-related toxicities developed, the subsequent cycle was delayed until the absolute neutrophil count was ≥1.0 × 10^9^/l, the platelet count was ≥100 × 10^9^/l, and the treatment-limiting toxic effect decreased to ≤ grade 1.

Tumor response was assessed using the Response Evaluation Criteria in Solid Tumors, version 1.1. PFS was measured from the first day of treatment on the clinical trial until the date of disease progression or death, whichever came first. Patients’ characteristics were analyzed using descriptive statistics. Spearman rank was used to analyze the correlation between demethylation change, platinum content changes, CTR1 cytoplasmic and nuclear scores, Ki-67 percentage, and the time on study. All *P* values presented are two-sided and statistical significance means *P* ≤ 0.05.

## Additional file

Additional file 1: Table S1. Characteristics of treated patients with stable disease.
