# Supplementary material for: Phase I study of azacitidine and oxaliplatin in patients with advanced cancers that have relapsed or are refractory to any platinum therapy
Source: Clin Epigenetics. 2015 Mar 17;7(1):29. doi: 10.1186/s13148-015-0065-5 (PMC4371799; doi:10.1186/s13148-015-0065-5)
Supplement: Additional file 1: Table S1. — Characteristics of treated patients with stable disease. [file 13148_2015_65_MOESM1_ESM.pdf]

Supplemental Table 1. Characteristics of treated patients with stable disease

Sex/Age	ECOG	Cohort	Number of prior therapies	No. of cycles	Type of cancer	RECIST, %	PFS
F/56	1	1	5	4	CRC	8	4.3
M/71	1	3	4	6	Prostate	4	5.7
F/45	1	6	5	4	Lung	-6	4.8
M/64	1	6	3	4	CRC	7	4.0
M/79	1	6	3	2	H&N	2	2.3
F/63	1	6	6	2	Endometrial	10	2.3
F/72	1	6	5	6	CRC	8	5.8
M/58	1	Ex	8	4	CRC	9	4.6
M/62	1	Ex	5	2	CRC	19	1.9
F/59	0	Ex	3	5	CRC	3	5.3
M/53	1	Ex	2	1	CRC	15	1.6
F/57	0	Ex	3	4	CRC	6	3.9
M/48	1	Ex	4	1	Lung	9	1.1

F; female, M; male, Ex; expansion phase, CRC; colorectal, H&N; head and neck carcinoma, PFS; progression-free survival.
